# Free viewing biases for complex scenes in preschoolers and adults

**DOI:** 10.1038/s41598-023-38854-8

**Published:** 2023-07-21

**Authors:** Marcel Linka, Özlem Sensoy, Harun Karimpur, Gudrun Schwarzer, Benjamin de Haas

**Affiliations:** 1 Department of Experimental Psychology, Justus Liebig University Giessen, 35394 Giessen, Germany; 2 Department of Developmental Psychology, Justus Liebig University Giessen, 35394 Giessen, Germany

**Keywords:** Human behaviour, Visual system

## Abstract

Adult gaze behaviour towards naturalistic scenes is highly biased towards semantic object classes. Little is known about the ontological development of these biases, nor about group-level differences in gaze behaviour between adults and preschoolers. Here, we let preschoolers (*n* = 34, age 5 years) and adults (*n* = 42, age 18–59 years) freely view 40 complex scenes containing objects with different semantic attributes to compare their fixation behaviour. Results show that preschool children allocate a significantly smaller proportion of dwell time and first fixations on *Text* and instead fixate *Faces*, *Touched* objects, *Hands* and *Bodies* more. A predictive model of object fixations controlling for a range of potential confounds suggests that most of these differences can be explained by drastically reduced text salience in pre-schoolers and that this effect is independent of low-level salience. These findings are in line with a developmental attentional antagonism between text and body parts (touched objects and hands in particular), which resonates with recent findings regarding ‘cortical recycling’. We discuss this and other potential mechanisms driving salience differences between children and adults.

## Introduction

To process visual information at high resolution, we constantly have to move our eyes. The periphery of the visual field suffers from crowding and lack of acuity, implying the need to prioritise certain areas in a scene over others^1,2^. How does the oculomotor system decide these priorities? Does it learn them over time? And if so, how do they change across the lifespan?

### Adult gaze behavior

The past 25 years have seen extensive attempts to predict adult gaze behaviour based on the content of a visual scene. Two types of scene features have emerged as particularly relevant, namely low-level features such as the local contrast in orientation, intensity and color^3,4^ and high-level objects such as faces and text^5^. Objects^6^ and their semantic features^5^ have been found to be substantially more predictive of gaze behaviour than classic low-level salience. For instance, objects^6,7^ and in particular those that are faces, text, touched objects and objects with implied motion^5,8^ hold large weights in predicting adult gaze behaviour, outweighing low-and mid-level features in complex scenes^5,9^. These attentional biases towards certain semantic features are shared between individuals, but their degree differs substantially and reliably^10,11^. Moreover, some gaze biases seem to be particularly pronounced among observers with specific perceptual abilities or challenges. For instance, patients with autism spectrum disorder (ASD) spend less time fixating faces and social features compared to controls^12,13^. Super-recognizers—people with exceptional abilities for processing facial identity information—spend more time fixating faces and less time focusing on touched objects and text^14^.

### The development of gaze behavior

While visual preferences for semantic features have been studied extensively for adults, much less is known about their development. A notable exception are faces. Infants show a visual preference for face-like dot patterns over inverted patterns very early on^15,16^. In fact, recent evidence points to increased behavioural responses to such face-like stimuli by human foetuses even during the third trimester of pregnancy^17^ (but note that this conclusion has been questioned on methodological grounds^18,19^). These findings contribute to a long-standing debate on the question whether human attentional biases towards faces are driven by innate mechanisms^16,20^. In the course of infancy, this attentional bias develops into a more differentiated preference for faces over mere face-like stimuli^21^ and non-face objects in complex scenes^22–24^. Aside from faces, infants show gaze biases towards other socially relevant stimuli. For example, Frank et al. let children from 3- to 30-months of age freely view videos of complex social scenes depicting children performing everyday actions (mostly playing with toys). They found that infants spent a larger proportion of their dwell time fixating hands when a depicted scene turned socially complex, that is, when agents in the videos used their hands for more complex actions^25^. This effect was positively correlated with age, suggesting that throughout infancy and early childhood, gaze allocation becomes increasingly tuned to socially relevant visual information. This bias for social information might go hand in hand with an early visual sensitivity for biological motion in (new-born) infants^26^. More recent work shows that a visual preference for implied motion in static stimuli might emerge already at around 5 months of age^27,28^. As with adults^10,29^, the degree of attentional biases towards social information varies reliably in childhood and is highly heritable^12,30^. Visual attention in infants is however not only dependent on the object’s semantics or the individuals’ gaze biases. Work using head camera recordings of infants and their parents playing in naturalistic scenes has shown that prior parental attention towards objects results in extended visual selection for a given object^31^. Interestingly, infant attention and even more so, joint attention towards objects during word learning predicted later vocabulary size in infants^32^. Fewer studies have investigated the development of attentional biases beyond three years of age.

Açık et al. have looked at free-viewing behaviour towards complex scenes across three age groups (7–9-year-old children, 19–27-year-old adults, and > 72-year-olds) and showed that the effect of low-level saliency on gaze drops with age^33^. Further, Helo et al. compared free-viewing behaviour of adults with those of children between 2 and 10 years and found that saccadic amplitudes increased and fixation duration decreased as a function of age^34^. Krishna et al. found that the center bias in image viewing decreases with age^35^. These studies suggest that gaze behaviour towards complex scenes changes from preschool/school age to adulthood, shifting from fixating more local, low-level features like luminance contrasts and orientation, to more global, explorative viewing behavior. However, more recent work predicting gaze behaviour in children (age 6–14) and adults showed that a face-based model outperformed (low-level) saliency models in predicting gaze towards complex scenes even in the youngest age group. The face-based model however was more predictive for gaze behaviour of adults than children^34^. This suggests that gaze behavior is more attracted to faces than low-level features and that this face preference increases with age.

Taken together, we know of distinct semantic information that guides viewing behaviour in adults and infants. Gaze behaviour in older children seems to differ significantly from adults in terms of enhanced low-level salience, center-bias, fixation durations and lower saccadic amplitudes^33,34^. However, just as for adults, the gaze behaviour of children is better predicted by high-level semantic information compared to low-level features^5,36^. Although we know of qualitative differences in the effect of semantic information on fixation behavior in adults and infants, it is unclear whether and how gaze behaviour towards semantic information differs between preschool children and adults.

Besides *Faces* and *Touched* objects, *Bodies* and *Hands* adults also show high (but individually variable) salience for *Text*^5,10^. To date, it is unclear how these biases for *Text* develop, whether they exist before reading acquisition and how they interact with other semantic gaze biases. Here, we probe this question by comparing free viewing biases for *Text*, *Faces*, *Touched* objects, as well as *Bodies* and *Hands* between preschool children and adults free-viewing complex scenes.

Understanding whether and how gaze behaviour in children is drawn towards semantic content can have important implications. Atypical gaze behaviour towards faces has been found in infants and children with ASD^37,38^. Insights on semantic gaze biases in healthy children may help establishing a baseline to test the diagnostic potential of gaze behaviour for neurodevelopmental disorders. Moreover, given the increasing exposure of children to screen-based education, understanding their attentional biases may help in the design of efficient learning material. Investigating visual attention in preschoolers is particularly relevant, because this age represents a time of remarkable psychological growth; For example, selective attention develops significantly during later childhood, especially between 4 and 7 years of age^39^. Further, during the preschool years, children have an innate ‘theory drive’, that is they ask many questions and actively seek causal explanations in order to interpret and understand things in their environment^40,41^. This and a highly dynamic neural development (also in the visual cortices^42,43^) make preschoolers a particularly relevant age group for studying visual attention towards complex scenes as insights may inform theories on neural and cognitive functioning.

### The present study

In the present study we investigated gaze behaviour towards 40 complex scenes in 5-year-old preschoolers and adults. In particular, we tested whether and to what extent children compared to adults differ in their proportions of (1) cumulative dwell time and (2) first fixations towards objects of multiple semantic categories depicted in these scenes, namely *Faces*, *Text* and *Touched* objects. First fixations have been described as driven by automatic or bottom-up processes^5,44–46^. Differences in first fixation allocation may thus reflect observer traits which are less malleable than those reflected in total dwell time.

Here, proportion of cumulative dwell time refers to the summed duration of all fixations falling on a given feature, divided by the summed duration of fixations falling on any object. First fixation proportions refer to the proportion of first fixations landing on any object, which landed on objects of a given category. First fixations refer to the first fixation initiated at least 100 ms after image onset (i.e. following the first saccade after image onset). Note that these proportions do not necessarily add up to 1. Any object-directed fixation can be labelled with none or multiple of the features analysed here. For example, a plate of pasta may bear none of the features analysed here, while text can be touched, too.

We chose these dimensions because of their importance for predicting adult gaze behaviour. Moreover, we have previously found that individual gaze tendencies along these dimensions can be tested reliably with this small stimulus set^5,10,11^. The recent publication of additional pixel masks and meta data for this stimulus set^47^ further allows the quantification of fixation biases towards *Bodies* and *Hands* depicted in the scenes. Adult gaze behavior is also predicted by objects with implied *Motion*. However, the moving objects in our stimulus set were almost exclusively person-related features (*Bodies*, *Hands* or *Faces*), rendering it ill-suited to estimate an independent contribution of this dimension. We did not consider *Motion* for our main analyses, but include a corresponding analysis in the Supplementary Material.

## Results

### Simple descriptive and inferential statistics

**Figure 1:**
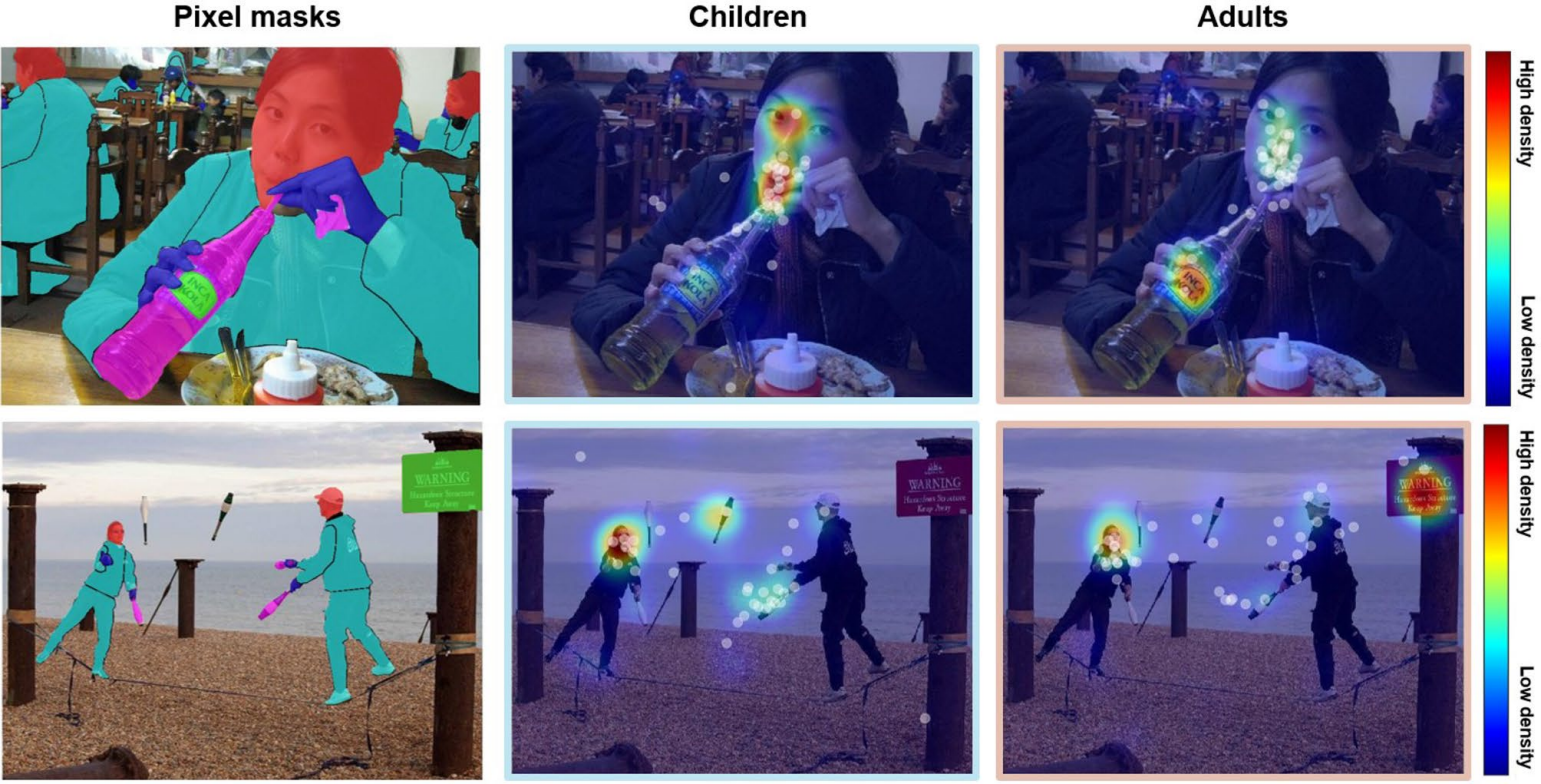
Pixel masks and heat maps showing group-wise fixations for two example images. Example stimuli on the left-hand side depict overlaid pixel masks for objects of the semantic dimensions: *Text* (green), *Faces* (red), *Touched* (violet), *Hands* (blue) and *Bodies* (cyan). Heat maps in the middle show corresponding duration weighted fixation data from children, maps on the right-hand side show fixations from adults. The transparent white scatters show all first fixations towards the image by all subjects of the respective group. Please note the increased gaze towards touched objects/hands and moving objects in the heatmaps for children, as well as the lower levels of text fixations compared to those of adults. Heatmaps and pixel mask overlays were created using MATLAB R2019B (MathWorks).

**Figure 2:**
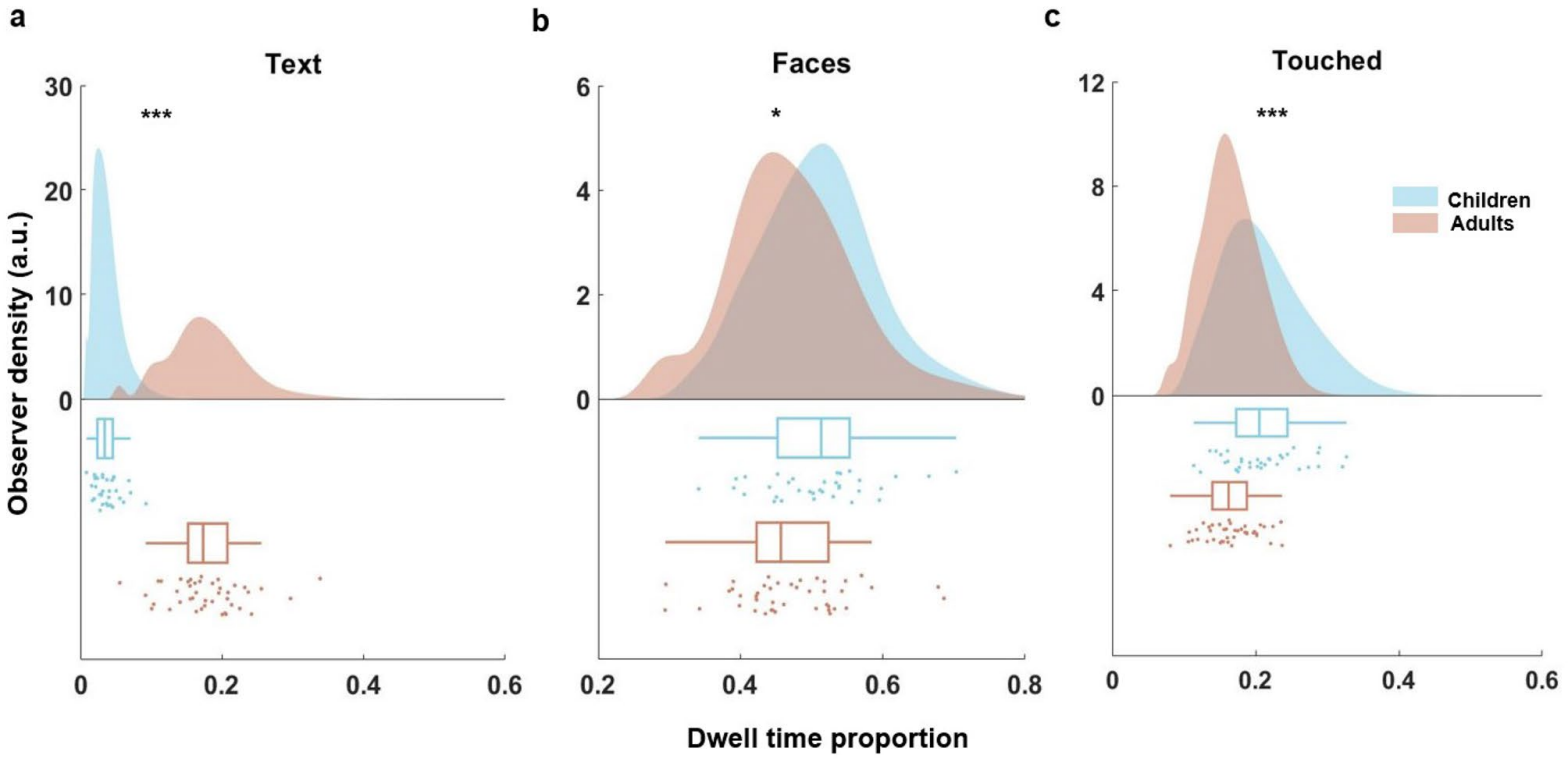
Differences in gaze behaviour along three semantic dimensions. Density plots showing the probability distribution of cumulative dwell time proportion towards objects of the dimensions *Face* (**a**), *Text* (**b**), and *Touched* (**c**) respectively for children (blue) and adults (red). Data points below the distributions indicate the individual dwell time proportion. Box plots depict the summary statistics for each dimension and group. For a respective box plot, the vertical line indicates the mean cumulative dwell time proportion. The left side of the box indicates the 25th percentile and the right side the 75th percentile. The whiskers represent the minimum and maximum values. *p < 0.05, **p < 0.01, ***p < 0.001 (Holm-Bonferroni corrected; see Methods).

First, we investigated whether children vs. adults show differences in gaze behaviour towards semantic information on a simple descriptive and inferential level. We analysed group differences in the proportion of cumulative dwell time and first fixations on *Text, Faces* and *Touched* objects. Figure 1 shows example heatmaps of cumulative dwell time for both groups as well as overlaid pixel masks. Figure 2 shows the distributions of cumulative dwell time proportions for children and adults respectively for each of the three semantic dimensions. See Table 1 for the mean pixel coverage of each semantic category.

**Table 1:** Mean % coverage of pixel masks per included semantic category

Pixel masks	Text	Faces	Touched	Bodies	Hands	Total
Coverage (mean %)	2.83	4.94	5.21	14.51	1.53	27.36
SD (across images)	2.3	5.09	5.45	13.8	1.8	17.11

Mean % of coverage represents the mean % of pixels covered in an image by objects of a given semantic dimension across all images depicting objects of a given dimension. SD gives the standard deviation of relative pixel coverage per semantic dimension across images depicting objects of a given dimension. Total includes the mean % and standard deviation (SD) of pixel masks of all 5 analysed dimensions across images.

#### Dwell time differences towards semantic categories

Results showed that the proportion of cumulative dwell time children spent on *Text* was reduced fivefold compared to adults, *t*obs = − 14.59*, p* < 0.001. Further, children spent a significantly larger proportion of their dwell time on *Faces*, *t*obs = 2.02*, p* = 0.02. and *Touched* objects, *t*obs = 4.28*, p* < 0.001 than adults.

#### First fixations towards semantic categories

**Figure 3:**
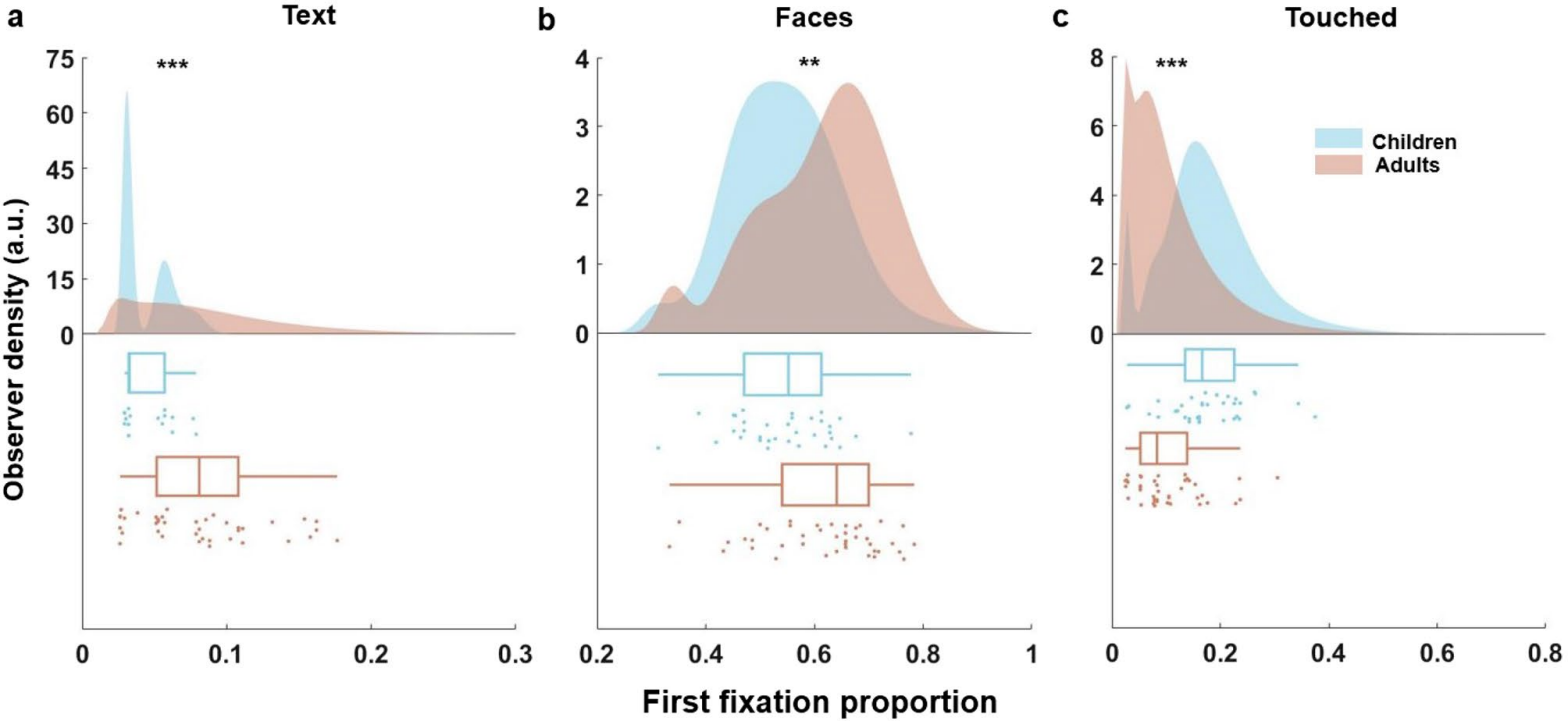
Differences between children and adults in first fixation proportion for three semantic dimensions. Density plots show the probability distribution of first fixation proportion towards *Faces* (**a**), *Text* (**b**) and *Touched* objects (**c**) respectively for children (blue) and adults (red). Box plots below show an overview of the summary statistics for each group and semantic dimension; the vertical line within a respective box represents the mean value. The left side of a box indicates the 25th percentile and the right side the 75th percentile. The whiskers represent the minimum and maximum values. Data points represent the individual corresponding first fixation proportions. *p < 0.05, **p < 0.01, ***p < 0.001 (Holm-Bonferroni corrected; see Methods).

In a further descriptive analysis, we tested whether children and adults differ in their proportion of *first* fixations towards *Text*, *Faces* and *Touched* objects, that is the proportion of immediate saccades after image onset landing on objects from these categories. Figure 3 shows the distributions of first fixation proportions for children and adults. Findings show that children directed a significantly smaller proportion of first fixations towards *Text, t*obs = − 5.76*, p* < 0.001 and *Faces*, *t*obs = − 3.02*, p* = 0.002 and a significantly larger proportion of first fixations towards *Touched* objects, *t*obs = 4.28*, p* < 0.001.

#### Gaze behaviour towards hands and bodies

**Figure 4:**
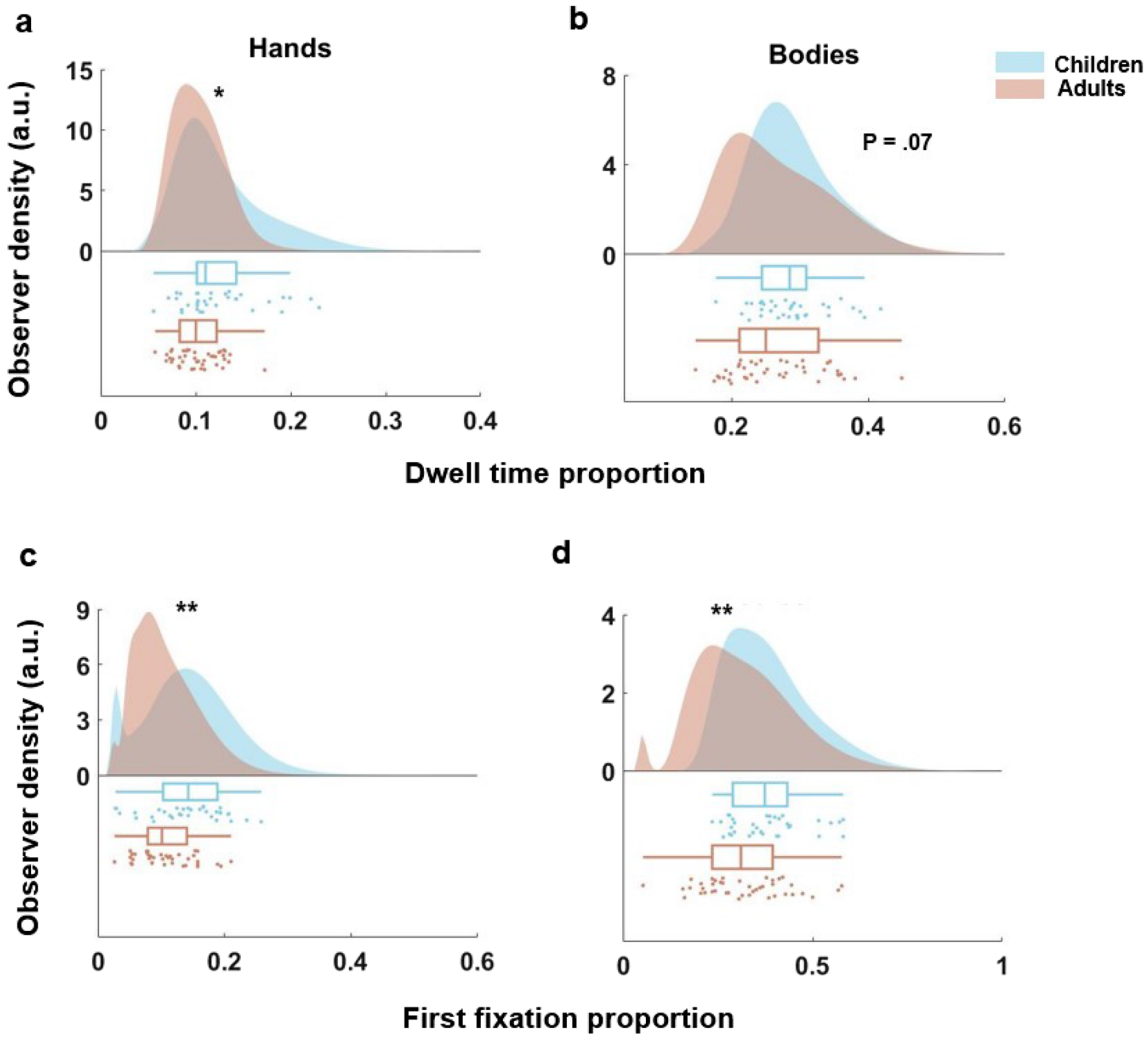
Group differences in gaze behavior towards hands and bodies. Density plots showing the probability distributions of cumulative dwell time (**a**, **b**) and first fixation proportion (**c**, **d**) towards objects of the dimensions *Hands* (**a**, **c**) and *Bodies* (**b**, **d**) for children (blue) and adults (red). Data points depicted below indicate the individual dwell time and first fixation proportion towards the respective dimension. Corresponding box plots above the data points show an overview of the summary statistics for each group and semantic category; the vertical line within a box represents the mean value. The left side of a box indicates the 25th percentile and the right side the 75th percentile. The whiskers represent the minimum and maximum values. *p < 0.05, **p < 0.01, ***p < 0.001 (Holm-Bonferroni corrected; see Methods).

Given enhanced fixation tendencies towards *Faces* and *Touched* objects in children and recent findings on fixation tendencies in adults^47^, we additionally tested whether similar effects would emerge for *Hands* and *Bodies* (with the latter excluding hands). Figure 4 shows the distributions of cumulative dwell time (a, b) and first fixation (c, d) proportions spent on *Hands* and *Bodies* for children and adults. Children did not significantly differ from adults in the proportion of dwell time spent on *Bodies* (although there was a trend for children to fixate more towards *Bodies*, *t*obs = 1.5*, p* = 0.07), but spent a significantly larger proportion of dwell time on *Hands* (*t*obs = 2.78*, p* = 0.01). Moreover, children directed a larger proportion of first saccades towards *Hands* and *Bodies* compared to adults (*Hands*: *t*obs = 2.83*, p* = 0.007, *Bodies*: *t*obs = 2.58*, p* = 0.007).

Note, for these analyses, we focussed on group differences in object-directed dwell time and first fixations. Although children and adults both spent the vast majority of their dwell time and first fixations on objects (rather than the background), we found that children did so significantly less (dwell time on objects for children and adults: *M* = 88% and 90%, respectively; *tobs* = − 1.98, *p* = 0.02; first fixations *M* = 86% and 92%, *tobs* = − 5.47, *p* < 0.001). Nevertheless, including background fixations in our analyses did not change the pattern of results. Results of the corresponding control analyses are reported in the supplementary materials.

### (G)LMM analyses

Adults may spend less time fixating *Faces*, *Touched* objects and *Hands* because of their higher propensity to fixate *Text*, or an inherently lower tendency to fixate these other dimensions. To juxtapose these hypotheses and control for explicit lower-level salience features^48,49^, we fitted a Linear Mixed Effect Model (LMM) using fitlme() from the Statistics and Machine Learning Toolbox in MATLAB R2019b. This model predicted the cumulative dwell time towards an object given its high-level semantic features as well as size, image eccentricity, and graph-based visual salience (GBVS)^3^, for which previous work has shown age-dependent differences^33,35,48^.

Moreover, we fitted a binomial Generalized Linear Mixed Model (GLMM) using the function fitglme() from the Statistics and Machine Learning Toolbox in MATLAB R2019b to estimate the probability that a given object was fixated immediately after image onset (first fixation), again, as a function of high-level semantic features, size, image eccentricity, and graph-based visual salience (GBVS)^3^. To show the influence of the group difference in Text fixations on gaze differences towards any other semantic category, we have additionally fitted trimmed (G)LMMs, excluding Text (see supplementary materials for results).

**Figure 5:**
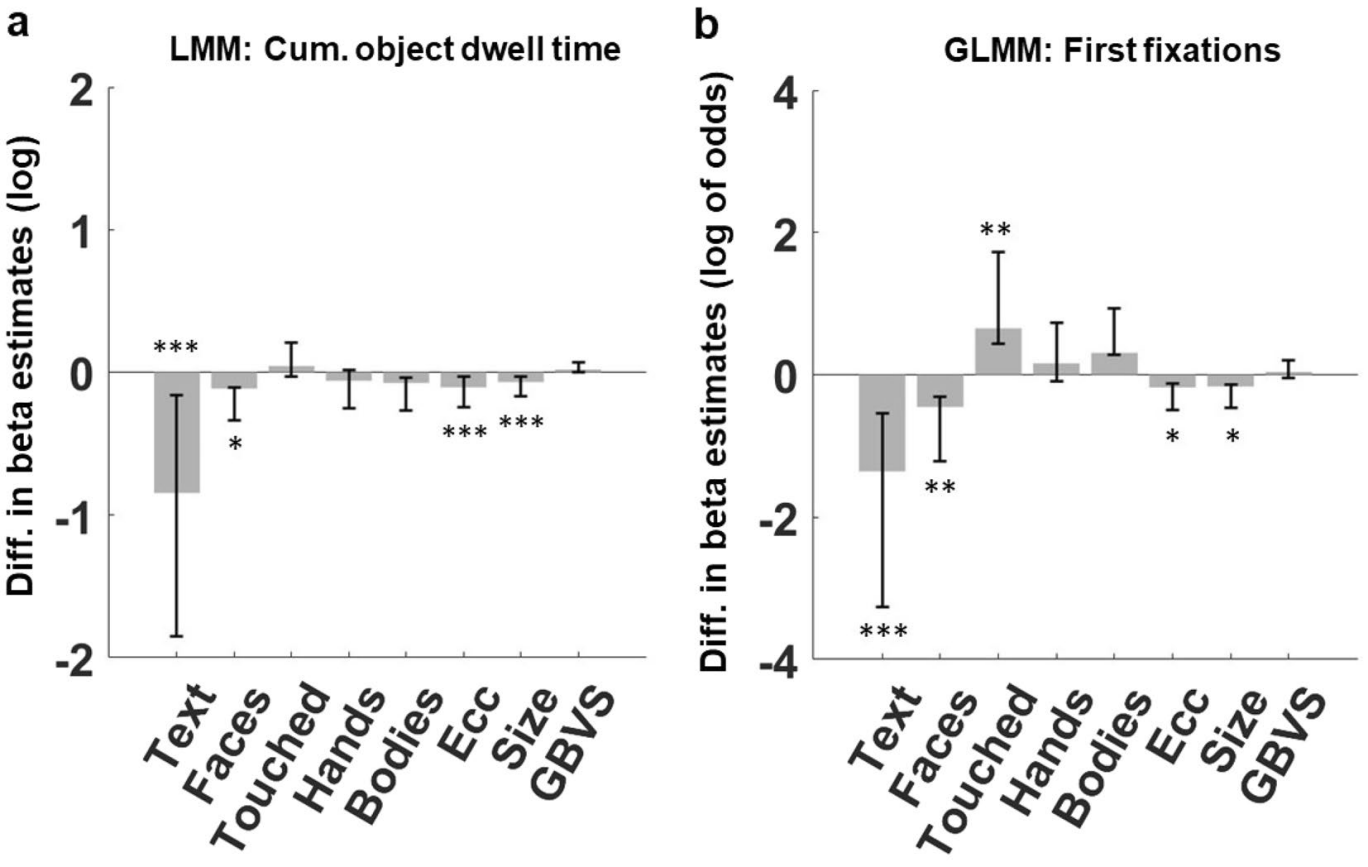
Group differences in predicting object-directed dwell time and first fixations. Bar plots depicting the beta estimates of a given object feature x *Age Group* interaction, which describe the difference in estimates between children and adults for a given object property. Panel a shows the interaction estimates for the LMM predicting object-directed dwell time and panel b depicts the binomial GLMM predicting first fixations towards objects after image onset across the included predictors *Text*, *Faces*, *Touched*, *Hands* and *Bodies* as well as Object-image centroid eccentricity (Ecc), (Object-) Size and graph-based visual salience (GBVS). Asterisks indicate the significance of a given group x predictor interaction. Results of (G)LMMs that did not take into account the effect of Text can be found in the supplementary materials. Error bars represent the 95% confidence intervals. *p < 0.05, **p < 0.01, ***p < 0.001.

Model coefficients, standard errors, and corresponding *t*-values and *p*-values for results are reported in Tables 2 and 3 respectively. Figure 5 shows the estimates for each included interaction for the (a) linear and (b) binomial model.

**Table 2:** Results of linear mixed-effects model predicting object dwell time.

Fixed effects	Description	B	SE	*t*	*P*			
Intercept	-	** − 0.28**	**0.05**	** − 5.42**	** < 0.001**			
Age group	Children-adults	**0.21**	**0.05**	**4.17**	** < 0.001**			
Text	Main effect	0.15	0.09	1.65	0.10			
Age group:text	Children-adults	** − 0.84**	**0.08**	** − 10.16**	** < 0.001**			
Faces	Main effect	**0.52**	**0.07**	**7.38**	** < 0.001**			
Age group:faces	Children-adults	** − 0.12**	**0.06**	** − 2.05**	**0.04**			
Touched	Main effect	**0.23**	**0.08**	**2.78**	**0.01**			
Age group:touched	Children-adults	0.04	0.06	0.71	0.48			
Hands	Main effect	0.06	0.08	0.70	0.49			
Age group:hands	Children-adults	** − **0.06	0.07	** − **0.87	0.39			
Bodies	Main effect	** − **0.04	0.07	** − **0.52	0.60			
Age group:bodies	Children-adults	** − **0.08	0.06	** − **1.31	0.19			
Eccentricity	Main effect	** − **0.04	0.03	** − **1.60	0.11			
Age group:eccentricity	Children-adults	** − 0.11**	**0.02**	** − 6.33**	** < 0.001**			
Size	Main effect	**0.26**	**0.02**	**11.00**	** < 0.001**			
Age group:size	Children-adults	** − 0.07**	**0.02**	** − 4.10**	** < 0.001**			
GBVS	Main effect	**0.09**	**0.03**	**3.21**	**0.001**			
Age group:GBVS	Children-adults	0.02	0.02	1.04	0.30			

Random effects								
Groups	Name	*SD*	*r*					
Subjects	Intercept	0.18	Intercept					
	Text	0.18	** − **0.03	Text				
	Faces	0.16	0.01	** − **0.19	Faces			
	Touched	0.14	** − **0.25	0.30	** − **0.59	Touched		
	Hands	0.17	0.04	0.29	** − **0.30	0.90	Hands	
	Bodies	0.16	** − **0.27	0.47	0.03	0.76	0.86	Bodies
Objects	Intercept	0.41	0.41					
Scenes	Intercept	0.07	0.07					

Differences between groups were tested by including the relevant interaction terms. A beta estimate for a given interaction represents the estimated difference between children and adults. See methods for information regarding the applied coding scheme. Main effects represent the average estimates across both age groups. Significant effects are reported in bold.

**Table 3:** Results of binomial generalized linear mixed-effects model predicting first fixations towards objects.

Fixed effects	Description	B	SE	*t*	*P*			
Intercept	-	** − 4.92**	**0.21**	** − 23.46**	** < 0.001**			
Age group	Children-adults	** − **0.08	0.14	** − **0.60	0.55			
Text	Main effect	0.18	0.37	0.48	0.63			
Age group:text	Children-adults	** − 1.35**	**0.28**	** − 4.83**	** < 0.001**			
Faces	Main effect	**2.38**	**0.28**	**8.35**	** < 0.001**			
Age group:faces	Children-adults	** − 0.46**	**0.16**	** − 2.91**	**0.004**			
Touched	Main effect	0.62	0.34	1.80	0.07			
Age group:touched	Children-adults	**0.65**	**0.22**	**2.99**	**0.003**			
Hands	Main effect	0.56	0.34	1.67	0.09			
Age group:hands	Children-adults	0.16	0.21	0.75	0.45			
Bodies	Main effect	0.24	0.29	0.83	0.41			
Age group:bodies	Children-adults	0.31	0.17	1.84	0.07			
Eccentricity	Main effect	** − 1.28**	**0.13**	** − 10.03**	** < 0.001**			
Age group:eccentricty	Children-adults	** − 0.18**	**0.07**	** − 2.68**	**0.01**			
Size	Main effect	**1.66**	**0.12**	**13.36**	** < 0.001**			
Age group: size	Children-adults	** − 0.17**	**0.07**	** − 2.48**	**0.01**			
GBVS	Main effect	**0.78**	**0.13**	**6.05**	** < 0.001**			
Age group:GBVS	Children-adults	0.04	0.06	0.60	0.55			

Random effects								
Groups	Name	*SD*	*r*					
Subjects	Intercept	0.18	Intercept					
	Text	0.18	** − **0.79	Text				
	Faces	0.16	0.72	** − **0.23	Faces			
	Touched	0.14	** − **0.57	0.06	** − **0.98	Touched		
	Hands	0.17	0.84	0.35	** − **0.81	0.73	Hands	
	Bodies	0.16	** − **0797	0.27	0.77	0.70	0.99	Bodies
Objects	Intercept	1.56	–					
Scenes	Intercept	0.0002	0.07					

Differences between groups were examined by including the relevant interaction terms. A beta estimate for a given interaction represents the estimated difference between children and adults. See methods for information regarding the applied coding scheme. Main effects represent the average estimates across both age groups. Significant effects are reported in bold.

The LMM's intercept holds no specific significance in this context, as it indicates the estimated average value (log-transformed and standardized) of object-directed dwell time when all predictors, including semantic variables are equal to zero. When referring to z-standardized continuous predictors, the value of zero corresponds to the average value of the predictor. The significant effect for *Age Group*, (*b* = 0.21,* t* = 4.17, *p* < 0.001), indicates that children spent more dwell time towards a selected object than adults. Note, this does not contradict the earlier finding that children spent less time fixating objects overall compared to adults (see simple descriptive and inferential statistics), because here we look at the average dwell time for a given object (children tend to look at fewer objects overall and therefore spent more time engaging with them). We also found a significant interaction between *Age Group* and *Text*, *b* = − 0.84, *t* = − 10.16, *p* < 0.001, indicating longer dwell time towards *Text* for adults compared to children. Further, we found a small but significant interaction between *Age Group* and *Faces*, *b* = − 0.12,* t* = − 2.05, *p* = 0.04, also indicating somewhat higher dwell time on faces for adults compared to children. No significant interactions were found between *Age Group* and *Touched*, *b* = 0.04*, t* = 0.71*, p* = 0.48, *Age Group* and *Hands*, *b* = − 0.06, *t* = − 0.87. *p* = 0.39, and *Age Group* and *Bodies*, *b* = − 0.08, *t* = − 1.31, *p* = 0.19, indicating no significant dwell time differences between children and adults for objects of these categories when controlling for the other dimensions.

Regarding lower level features, we found a significant interaction between *Age Group* and *Eccentricity*, *b* = − 0.11, *t* = − 6.33, *p* < 0.001, indicating a stronger center-bias for children vs. adults. Finally, we found a significant object *Size* x *Age Group* interaction, *b* = − 0.07, *t* = − 4.10, *p* < 0.001, indicating a smaller effect of object *Size* on the dwell time of children compared to adults. No significant interaction was found between *Age Group* and *GBVS*, *b* = 0.02, *t* = 1.04, *p* = 0.30.

We also estimated the probability that a given object attracted the first fixation after image onset. Again, the intercept bears no relevant information as it represents the probability of first fixating on an object that lacks any of the incorporated semantic labels, while exhibiting average values across all other continuous variables. The non-significant effect for *Age Group* (*b* =  − 0.08, *t* = − 0.60, *p* < 0.55) indicates a similar probability to first fixate an object across age groups. The probability that *Text* attracted the first fixation was smaller for children vs. adults, *b* = − 1.35, t = − 4.83, *p* < 0.001 and the same was true for *Faces*, *b* = − 0.46, *t* = − 2.91, *p* = 0.004. Further, a significant interaction was found between *Age Group* and *Touched* objects, *b* = 0.65, *t* = 2.99, *p* = 0.003, indicating a larger probability for *Touched* objects to attract the first fixation in children compared to adults. No significant interactions were found between *Age Group* and *Hands*, *b* = 0.16, *t* = 0.75, *p* = 0.45, and *Age Group* and *Bodies*, *b* = 0.31 *t* = 1.84, *p* = 0.07. Regarding lower level features, larger objects had a higher probability to attract first fixations in adults compared to children (*Age Group* x *Size* interaction; *b* = − 0.17, *t* = − 2.48, *p* = 0.01, and children showed a stronger center-bias for first fixations (negative effect of object eccentricity) *b* = − 0.18, *t* = − 2.68, *p* = 0.01. Finally, no group difference was shown for object-based low-level salience (*GBVS*), *b* = 0.04, *t* = 0.60, *p* = 0.55.

## Discussion

Adult gaze behaviour towards complex scenes is strongly biased towards semantic object categories^5^ and individual observers show large and reliable differences in the magnitude of these biases^10^. Many of these biases appear early on: Infants already show looking preferences for faces^15^, hands^25^ and (implied) motion^26–28^. At the same time, the fixations of adults free-viewing complex scenes are highly drawn to text elements, which pre-literate children may attend less. Here, we investigated the gaze behavior of preschool children towards complex scenes and tested their biases for *Text*, *Faces*, *Hands*, *Touched* objects and *Bodies*.

In our simple inferential statistics, we show substantial differences in attentional biases between children and adults. Compared to adults, children spent a larger proportion of dwell time on *Faces*, *Touched* objects*, Hands* and (albeit only trending) *Bodies* when freely viewing complex scenes. Most significantly, children spent a lot less time fixating *Text*. Moreover, we found that children compared to adults placed significantly fewer first fixations on *Faces* and *Text*, but more on objects being *Touched, Hands* and *Bodies*. To control for the interdependence of gaze biases – in particularly the strong bias differences for *Text*, as well as potential differences regarding the effect of lower-level features (object size, eccentricity and visual salience), we applied follow-up (G)LMM analyses. For the fully specified model predicting dwell time, we saw a substantial reduction in the dwell time attraction of *Text* and a slight reduction in the attraction of *Faces* in children vs. adults. When predicting the probability of first fixating an object of a given category after image onset, we found a reduced attraction of *Text* and *Faces* in children compared to adults, which was accompanied by an enhanced attraction towards *Touched* objects in children. Further, our findings indicate that children tend to spend less time looking at larger objects and are less likely to fixate on them first compared to adults. On the other hand, children have a stronger center bias, both in terms of their dwell time towards objects and their initial fixation than adults. Excluding the *Text* dimension from the models, however, resulted in enhanced weights for almost all person-related features in children than adults, except *Faces* (see supplementary results). This is in line with the idea that many descriptive gaze differences between children and adults can be explained by the drastically reduced tendency to fixate text in children. Children appear to allocate the fixations adults spent on text on person features and specifically on objects close to the hands.

For the main analyses, we did not include the semantic category *Motion* because there was an almost perfect overlap between body features (*Hands*, *Faces* and *Bodies*) and implied motion. An additional analysis suggests that children devote a significantly larger proportion of their dwell time towards objects with implied *Motion* (see supplementary materials). Future research should aim to test this motion bias in children in a stimulus set that allows to disentangle it from gaze biases towards body features.

We also found an enhanced center-bias in preschoolers, which matches recent evidence showing an enhanced center-bias in younger vs. older children^35^. Finally, we found no group difference in graph-based visual salience (GBVS) when predicting object-directed gaze behaviour. This contrasts previous reports of decreasing effects of visual salience with age^33^. One possible reason for this may be that the apparent group difference in the effects of visual salience may be better explained by semantic features, though this cannot be settled without targeted experiments explicitly disentangling the two (cf.^7^). Future research could test this explicitly.

Together, these findings contribute to our understanding of how human attention changes across the lifespan. Already very early in life our gaze becomes increasingly drawn towards distinct semantic social information^25^. This semantic information still seems to hold weight for preschoolers as well as adults. At the same time, our findings show that semantic gaze tendencies change between preschool childhood and adulthood, to include text, at the expense of attention towards body-related features like hands, bodies and objects being touched. This suggests differences in how adults and children perceive the same complex scenes, which in turn seem tied to the acquisition of reading. Interestingly, most of these changes in semantic attention extend to the first saccade after image onset. One exception are faces—*more* first fixations land on faces for adults compared to children. Whether this change of gaze biases appears as soon as literacy begins to evolve or gradually changes with reading experience is still an open question and represents an interesting subject for future research. Gaze behavior towards semantic features appears to be the result of an interaction between image content, individual differences and experience.

This may explain why heritability estimates for gaze behaviour towards social scenes are substantially higher for infants^12^ compared to older children (9–14 years^30^). Individual differences at the beginning of life seem dominated by genes, but may be modulated substantially by later experience, such as the acquisition of reading.

We hypothesize that reduced gaze behaviour towards text in children is due to limited literacy. Our preschool sample had not yet acquired formal education in reading and text features consequentially are uninformative to them. With age and improving reading ability, gaze behaviour may increasingly be tuned towards text at the expense of other semantic information. However, we did not assess reading ability so cannot exclude the possibility that at least some children already acquired reading skills before entering school.

As mentioned above, our basic inferential statistics further suggest that children spent a somewhat higher fraction of their dwell time on faces, but a *lower* fraction of their first fixations. This adds to previous works showing that face-based models are more successful in predicting gaze behaviour in adults compared to children^36^, but that children showed enhanced proportions of first fixations towards social information when memorising a scene^50^. Our LMM control analyses, which excluded text as an explanatory variable, did not indicate any significant difference in the dwell time attraction of faces between children and adults. This suggests that any observed preference for faces in children may be attributable to corresponding object features such as eccentricity, size, and GBVS. However, when we controlled for text, our model did show a significantly lower preference for faces in children compared to adults. The finding that children directed a *lower* fraction of first fixations towards faces extended to the GLMM controlling for all other factors and may point to two separate visual processes with dissociable developmental trajectories driving face salience during the earlier and later stages of viewing a scene: one, indicated by first fixation distributions, that is largely bottom-up, guiding attention immediately after stimulus onset and showing a stronger, possibly more matured bias towards faces in adults. This may reflect more accurate and faster face processing abilities in adults^46^, enabling them to saccade towards faces in the periphery very rapidly^46^. And a second one indicated by dwell time distributions, that may be under more top-down control and reflect the stronger competition of text with other semantic categories in adults for our stimuli.

Touched objects attracted a higher fraction of dwell time and first fixations in children compared to adults. The latter effect persisted in a GLMM controlling for all other modelled high- and low-level features. In a recent study, Nordt et al. showed that from young childhood to teen age, expanding word selective regions in ventral temporal cortex are directly linked to decreases in limb selectivity^43^. These findings are interpreted as evidence of cortical recycling of limb-selective areas for the visual word form area, emerging during reading acquisition. It is tempting to speculate that such effects of cortical recycling may be linked to the matching changes in visual preferences we see here—particularly for first fixations, that is, a shift in visual attention from limbs to text in adults compared to children.

Another candidate hypothesis for why children overall spent more of their dwell-time on touched objects and hands than adults is a possible immaturity of action understanding and social processing abilities. Critical social skills like reasoning about other people’s thoughts (theory of mind; ToM) likely continue to develop beyond 5 years of age^51–53^. Children may compensate for limited action understanding and social processing abilities by allocating more dwell time towards hands and touched objects to extract the information necessary for making sense of goals and behaviours of people depicted in the scenes. This may be particularly pronounced for briefly presented complex scenes including multiple objects, people and their interactions. This is underscored by our model-based results which suggests that early visual preference for touched objects is increased in children, even when controlling for the lower visual preference for Text.

Further, there is evidence that children are embodied learners, that is, they seem to build their knowledge more strongly on sensorimotor experiences compared to adults^54^. It is possible, that gaze preferences in children for hands and objects being touched reflect such tuning towards physical and motor experiences.

Finally, children’s enhanced gaze biases towards touched objects and hands may be related to their distinct drive to explore why and how things in the world interact as they do. Children seem to have a strong preference for understanding the causal structures surrounding them. For instance, when exposed to events that are inconsistent with their prior knowledge, preschool children seek causal explanations^55^. Moreover, preschoolers most frequently asked questions about functions and causal properties when they encountered novel objects^56,57^. This preference for causal information seems to be particular pronounced in preschoolers^58^. Children’s gaze biases towards objects being touched and hands could be a consequence of this early drive to understand cause and effect and the functionality of objects.

Future studies could test this hypothesized relationship between causal inference and corresponding eye movements in preschool children in controlled experiments, juxtaposing stimuli with well-known and novel functions for children. Further, one could compare gaze behaviour towards semantic information in preschoolers and children who have already experienced formal education to test the effect of literacy on attention and specifically whether it is gradual or sudden. Given recent evidence for cortical recycling during childhood^43^, future research could also examine whether a shift from cortical selectivity for limbs towards enhanced selectivity for words co-occurs with corresponding gaze biases. Finally, future research could probe gaze behaviour towards semantic dimensions in preschool children using more naturalistic stimuli, like videos of everyday scenes. This could reveal whether present gaze differences—possibly including implied motion—also hold for dynamic scenes.

Taken together, we report several differences in gaze biases between preschool children and adults. Children spent a far smaller proportion of dwell time on *Text* and more on *Faces*, *Touched* objects, *Hands* and a corresponding trend for *Bodies*. For the dimensions *Text*, *Touched* and *Hands*, these biases already show for first fixations after image onset, while children directed *fewer* first fixations on *Faces*. Further analyses controlling for *Text* and other salience factors (GLMM analyses) suggest that most body-related attentional biases in children are due to the lack of competition with *Text* seen in adults. One exception is the higher propensity of *Touched* objects to attract first fixations in children, which persisted even when controlling for *Text* salience.

These findings show that fixation behaviour towards semantic features changes over the lifespan. Children and adults may perceive the same visual environment in different ways, which in turn may be related to the acquisition of reading ability, socio-cognitive development and cortical recycling. Future research examining the relationship between these factors and gaze behavior can inform theories on the development of human visual attention.

## Methods

### Subjects

In total n = 47 children and n = 46 adults with normal or corrected vision took part in the study. We only included subjects with at least one fixation for more than 80% of the trials (34 trials). Applying this criterion, we removed 17 subjects from the original sample (13 children and 4 adults). This resulted in a final sample of *n* = 34 children and *n* = 42 adults.

Of the remaining sample, children on average showed 2.9% and adults 1.1% missing trials.

All subjects provided written informed consent before participation. For children, parents gave written informed consent. The study was approved by the local ethics committee of Justus-Liebig-University Giessen and adhered to the declaration of Helsinki.

Children (*n* = 34; *M*_age_ = 5,7; range = 5.1–5.9; *SD* = 0.17; 19 females) were recruited as part of a larger study based on a local data base of parents, who indicated their interest in child development studies. No child in the sample attended school at the time of the study. All children completed another task which was not related to the present study. Subjects received no financial reimbursement for participation. Children were rewarded with a certificate of participation and a small gift.

Adults (n = 42; *M*_age_ = 24.4; range = 18–59; *SD* = 7.05; 31 females) were recruited as part of a larger study and completed other tasks which were unrelated to the present study. Adult participants were compensated with money (7€/hr) or course credit for participation.

### Apparatus

The free viewing task was created and implemented using Psychopy 2020.1.2^48^ in Python version 3.6.10^59^. Stimuli were shown on a Lenovo ThinkPad X230 laptop with a screen resolution of 1366 × 768 pixels. The stimuli were presented with a size of 1000 × 750 pixels, roughly corresponding to 21 × 16 degrees visual angle (d.v.a.). Eye movements were recorded from both eyes using a head-free Tobii 4c Eye Tracker (Tobii AB, Danderyd, Sweden) operating in remote mode at 90 Hz and a distance of 50–90 cm. No official information has been released stating the accuracy or precision of the Tobii 4c Eye Tracker. Work evaluating the predecessor model Tobii EyeX has reported an accuracy of < 0.6° and precision of < 0.25°^60^.

### Stimuli and procedure

We used the OSIE40^5,11^ dataset, which includes 40 complex everyday scenes and corresponding pixel masks for 364 objects with binary labels for 12 semantic object dimensions. Additionally, we used pixel masks for bodies and hands recently published by Broda and de Haas^47^. We recently found that individual gaze tendencies towards semantic features could be estimated reliably with 40 images^11^. As in previous analyses^11^, the 12 labels for the semantic dimensions were modified in order to reduce overlap between them in the following way: The *Face* label was removed from all object fixations with the *Emotion* label; the *Smell* label was removed from all object fixations with a *Taste* label; and the *Operable* and *Gazed* label were removed from object fixations with a *Touched* label. Objects were labelled as *Touched*, when they were touched by a human or animal in the scene. The *Watchable* label was removed from all object fixations with the label *Text.*

The experiment took place at a laboratory with only the participant and experimenter present. All participants were placed at a distance of 50–60 cm from the screen/eye tracker (the recommended user distance of the eye tracker is between 50 and 90 cm) and reminded to sit as still as possible during the session. To assure an appropriate position, a custom position guide interfaced with the eye tracker and gave online feedback about whether or not a participant was placed within the trackable range. On average, subjects sat at a distance of ~ 54.5 cm from the screen (children = 57 cm, adults = 52 cm). All viewing angles were calculated based on the average position distance of the respective group.

After completing a child-friendly 5-point calibration and validation procedure, subjects were instructed to freely view 40 images. Each image was presented centrally on the screen for 3 s, with a fixation cross in between trials presented centrally on the screen. A trial could only be initiated if a subject’s gaze did not deviate 2 d.v.a. from the fixation cross for 1 s. Images were presented in the same order across subjects.

To test eye tracker accuracy across trials, we calculated the deviation of trial onset fixations from the nominal center for each individual and group (i.e. the median distance in d. v. a. between the fixation cross shown between trials and the onset fixation). Results indicate that this deviation was minimal for both groups (M = 0.37, SD = 0.14 for children and M = 0.46, SD = 0.23 for adults). The slightly larger error for adults was not statistically significantly different from that for children, *tobs* = 1.08, *p* = 0.07. To test the estimated drift across the task, we compared the deviation from the nominal center between the first and last trial for each observer and group (again in d.v.a.). We found that for children, there was no significant difference in drift between the first and last trials (M = 0.1, SD = 1.44, *tobs* = − 0.38, *p* = 0.34). For adults, there was a significant difference showing larger drift (M = 0.29, SD = 0.81, *tobs* = 2.38, *p* < 0.001). Finally, we tested the difference in drift between groups and found no significant differences (*tobs* = − 0.74, *p* = 0.07).

### Data processing

All pre-processing steps and statistical analyses were computed using MATLAB R2019B (MathWorks). Gaze data from both eyes were averaged. Fixations were extracted from raw eye tracking data by applying an inter-sample saccade velocity threshold of 30 d.v.a/s and a spatial inter-sample distance threshold of 2 d.v.a. Thus, samples were successively marked as fixations if both the corresponding inter-sample velocity was below 30 d.v.a/s and the spatial inter-sample distance was below 2 d.v.a. The latter criterion was applied to account for possible intermittent sample loss. Further, we used the median x and y position of samples deemed to be part of a fixation to determine the respective fixation location. Fixations with a duration < 100 ms were not considered. We further removed all fixations with an onset time (after trial onset) of < 100 ms to exclude fixations and saccades initiated before image onset. Saccade onsets and offsets were detected by applying an inter-sample velocity threshold of 30 d.v.a./s. Only saccades with amplitudes > 2 d.v.a. were considered.

**Figure 6:**
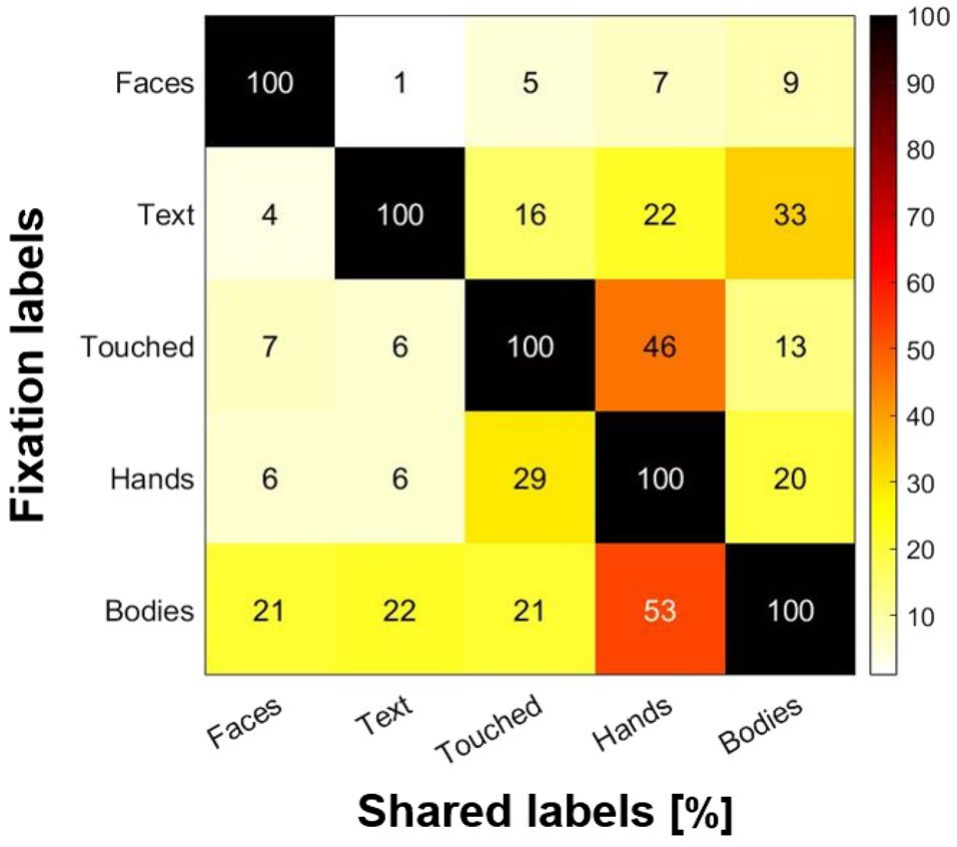
Label overlap across semantic categories. Heatmap showing the percentage of mutual label assignments. Each row shows the shared labels for all fixations bearing a given label. For example, the bottom row shows which percentage of *Bodies* fixations were also labelled as *Face*, *Text*, *Touched* and so on. Note, here we have calculated the proportion of fixations of a given category that is also labelled another category either (1) because the objects have several labels or (2) the fixation fell on several objects of different labels. The heatmap was created using MATLAB R2019B (MathWorks).

A given fixation can be assigned multiple semantic features. This is because we took the extent of the fovea and accuracy limits of the eyetracker into account by applying a tolerance margin when assigning semantic labels to fixations. Each fixation was assigned the labels of all objects falling within a radius of ~ 0.5 d.v.a. surrounding the pixel coordinates of the fixation. The margin size was selected based on the tested accuracy of the Tobii 4c eyetracker and its previous model. For more information on the eye tracker's accuracy, please refer to the information provided above. Additionally, for further discussion on best practices, please refer to Holmqvist et al.^61^ and Orquin et al.^62^*.* Figure 6 gives an overview of label overlap among all included fixations.

### Fixation tendencies across semantic dimensions

First, we tested differences in fixation tendencies along three semantic dimensions (*Faces*, *Text* and *Touched*) between children and adults. We chose these three dimensions, because adult gaze behaviour has been shown to reliably vary along them for the stimuli used in the present study (OSIE40)^11^. We calculated the proportion of individual cumulative dwell time falling on each of the aforementioned categories, which we previously found to be the best predictors of individual and group-level gaze behaviour. For this, the sum of respective fixation durations was divided by the sum of fixation durations on any object labelled in the OSIE40 or the *Body* and *Hand* masks which we derived from Broda & de Haas^47^. We further determined the proportion of all object directed first fixations (following the first saccade after image onset) which landed on *Text*, *Faces*, *Touched* objects for each participant (first fixation proportion. Given that some variables of interest were not normally distributed we tested group differences by applying non-parametric permutation tests. For this approach, in each iteration, we first pooled individual fixation data across all included 76 observers and then drew two random subsamples of respectively n = 34 and n = 42 without replacement from the pooled sample. After this, we calculated the mean difference between both samples for a given metric. This was repeated 10,000 times, resulting in a null distribution consisting of 10,000 permutated estimates. The *p*-value was determined as the proportion of permutated estimates that were more extreme than the true mean difference. Finally, we calculated the *t*-test statistic between the observed scores of both groups for a given metric (here referred to as *t*obs). *P*-values were Holm-Bonferroni for the number of included dimensions in each model. All group comparisons (Figs. 2, 3, 4) are visualized using Raincloud Plots^63^.

### Explaining object-directed fixations via (G)LMMs

Finally, we used (G)LMMs to explain the likelihood and cumulative duration of fixations as a function of object and observer properties. This allowed us to test age group differences in the predictive weights of semantic features, object size, eccentricity, and low-level salience. Our analysis followed the approach by Nuthmann et al., which employed a comparable model-based methodology while also accounting for center bias, object size, and visual salience^48,49^. Cumulative fixation duration towards objects was modeled as a continuous dependent variable of a LMM (estimated via Restricted Maximum Likelihood; REML). Here, cumulative fixation duration is defined as the total dwell time spent on a given object. Further, we only included objects that were fixated at least once. First object-directed fixation probability after image onset was modelled by fitting a binomial GLMM (using Laplace approximation) aiming to explain a binary response variable indicating whether an object was first fixated (1) or not (0) after image onset. Both models included the following fixed-effects terms: object-image *Eccentricity*, expressed as the distance between an object centroid and the image center in d.v.a.; object *Size* as the pixel coverage of an object in % of the corresponding image; the mean of graph-based visual salience (*GBVS*) across pixels of the object; and binary dummy coded variables indicating whether the object was labelled as *Text*, *Face*, *Touched*, *Hands* or *Body*. Finally, we included the predictor *Age Group*, using effect coding (− 0.5/0.5) with adults as the reference group. This has the advantage of retrieving main effects (average estimates across both age groups) for each given predictor variable instead of simple effects. Note that our interest here was limited to group comparisons and estimates have to be interpreted as relative to other types of objects (and differ from pixel-wise salience models in that regard). The continuous variables *Eccentricity*, object *Size*, *GBVS*, and fixation duration were z-standardized across all objects in the dataset. Moreover, we log-transformed object *Size* and cumulative fixation time, because both distributions were positively skewed. To test age group differences, we further included interaction terms between *Age Group* and each remaining predictor.

In our present study design the random factor *Subject* is nested under *Age Group*. Moreover, the random factor *Object* is nested under the random factor *Scene*. We therefore included *Subject* as random intercept as well as by-subject random slopes for all semantic feature predictors (*Text*, *Faces, Touched, Hands and Bodies*). Finally, we included *Object* and *Scene* (i.e. image) as random intercepts.

The formula for the model-based analyses for both the binomial as well as the linear model was:

Response variable ~ 1 + Age Group + Text + Age Group:Text + Faces + Age Group:Faces + Touched + Age Group:Touched + Hands + Age Group:Hands + Bodies + Age Group: Bodies + ObjSize + Age Group:ObjSize + Ecc + Age Group:Ecc + GBVS + Age Group:GBVS + (1 + Text + Faces + Touched + Hands + Bodies |Subject) + (1|Object) + (1|Scene).

## Supplementary Information

Supplementary Information.

## Data Availability

Anonymized data and code to reproduce the presented findings and figures are available at osf.io/78aqf/.
