# Supplementary material for: Free viewing biases for complex scenes in preschoolers and adults
Source: Sci Rep. 2023 Jul 21;13:11803. doi: 10.1038/s41598-023-38854-8 (PMC10362043; doi:10.1038/s41598-023-38854-8)
Supplement: Supplementary file 1 — Supplementary Information. [file 41598_2023_38854_MOESM1_ESM.docx]

**Supplementary Materials**

**Methods**

**Group differences in basic oculomotor metrics**

To test whether children and adults differ in their gaze behaviour along basic oculomotor measures, we computed the mean fixation duration, saccadic amplitude, fixation duration of first fixations, saccadic amplitude of first saccades and first saccade latency respectively for each individual and group. After this, we applied two sampled *t*-test for group comparisons. The corresponding *p*-values were Holm-Bonferroni corrected for all 5 tests. We recommend interpreting these results with caution, given the limited temporal resolution of the eyetracker we used (90 Hz).

**Group differences in gaze behaviour towards semantic dimensions**

Our main analyses excluded background fixations. To control for possible effects of this, we ran a control analysis testing group differences in the proportion of *overall* dwell time and first fixations towards *Text*, *Faces*, *Touched* objects, *Hands* and *Bodies*. All group differences were tested for statistical significance via permutation tests. *P*-values were Holm-Bonferroni corrected for the dimensions included in a given model.

**(G)LMM analyses not controlling for text**

To illustrate the influence of the group difference in *Text* fixations on gaze differences towards any other semantic dimension, we have additionally fitted trimmed (G)LMMs. These models predicted object-directed dwell time (LMM) and the probability of first object-directed fixation (binomial GLMM) based on all previously added predictors excluding *Text*.

**Group difference in dwell time towards objects with implied motion**

Finally, we tested group differences in cumulative dwell time towards objects with implied *Motion*. We did not include *Motion* in our main analyses, because it almost perfectly overlaps with *Bodies*, *Hands*, and *Faces*. Here we nevertheless explored the proportion of object-directed cumulative dwell time for *Motion* between children and adults and tested the group difference via a permutation test.

**Results**

**Group differences in basic oculomotor metrics**

Table S1 provides an overview of the mean values and standard deviations regarding all five included metrics for children and adults as well as test statistics and *p*-values for each group comparison. We found no significant group differences between children and adults for the variables mean fixation duration, mean saccadic amplitude, mean first fixation duration and first saccade latency. However, we found that children exhibited significantly larger first saccades compared to adults.

Table S.1. Group differences in basic oculomotor metrics

|  | Adults | |  | Children | |  |  |  |
| --- | --- | --- | --- | --- | --- | --- | --- | --- |
|  | *M* | *SE* |  | *M* | *SE* | *t* | *df* | *P* |
| Fix. duration (ms) | 302.6 | 11.18 |  | 318.41 | 9.48 | -0.05 | 74 | 0.63 |
| Sacc. ampl (DVA) | 5.87 | 0.12 |  | 5.68 | 0.11 | -1.11 | 74 | 0.27 |
| First fix. duration (ms) | 297.57 | 18.12 |  | 300.14 | 11.23 | -0.11 | 74 | 0.91 |
| First sacc. amplitude (DVA) | 3.96 | 0.08 |  | 4.46 | 0.16 | 2.91 | 74 | 0.004 |
| First sacc. latency | 357.7 | 25.5 |  | 377.12 | 11.77 | -0.64 | 74 | 0.52 |
|  |  |  |  |  |  |  |  |  |
|  |  |  |  |  |  |  |  |  |

**Group differences in gaze behaviour towards semantic dimensions including background fixations**

Fig. S1 shows the distributions and mean values of dwell time (a) and first fixation proportions (b) for each semantic dimension and group when including background fixations in the analysis. The pattern of results was highly similar to that observed when excluding background fixations (cf. Fig. 3 and 4, main text).

Figure S1. **Group differences in attentional biases towards semantic dimensions**. Bar plots indicate mean values for the proportion of overall dwell time (a) and proportion of overall first fixations (b) respectively for children (blue) and adults (red). Data points indicate the individual mean values for a given dimension and group. Error bars represent the SEM. **p* < 0.05, ***p* < 0.01, ****p* < 0.001 (Holm-Bonferroni corrected; see Methods)


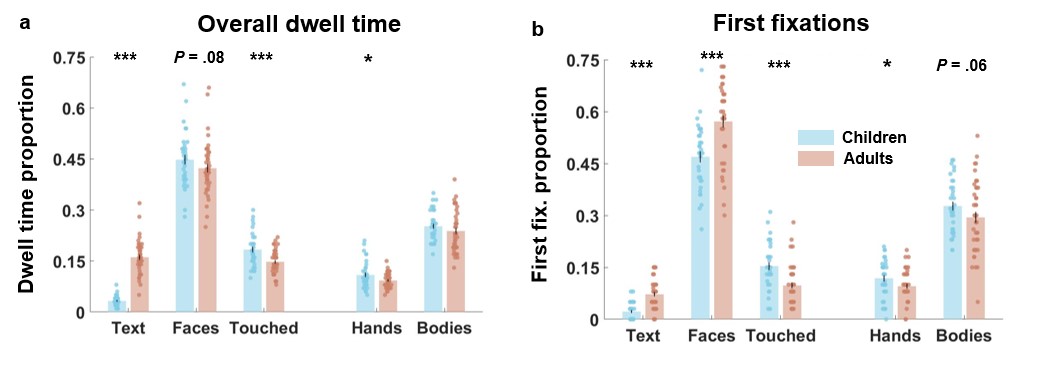


Our findings show that children spent a larger proportion of their overall dwell time towards *Touched* objects, *t*obs = 3.76*, p* < 0.001, *Faces* (only trending; *t*obs = 1.37*, p* = 0.08) and *Hands* (*t*obs = 2.25*, p* = 0.03). Further, children spent a lot less time fixating *Text* objects compared to adults, *t*obs = -14.36*, p* < 0.001. No significant group difference was found for the dimension *Bodies,* *t*obs = 1.1*, p* = 0.14*.* Considering group differences in first fixation proportions, we found that adults compared to children devoted a significantly larger proportion of their first fixations towards *Faces,* *t*obs = -4.22*, p* < 0.001 and *Text,* *t*obs = -5.98*, p* < 0.001. Further, children directed a significantly larger proportion of first fixations towards *Touched* objects, *t*obs = 3.92*, p* < 0.001. Finally, children showed a larger proportion of first fixation on *Hands,* *t*obs = 2.03*, p* = 0.047 and *Bodies,* *t*obs = 1.53*, p* = 0.06 (only trending).

**(G)LMM analyses not controlling for text**

Model coefficients, standard errors, *t*-values and *p*-values for results are reported in Tables S2 and S3 respectively. Fig S2 shows the estimates of each included Age group x object feature interaction for both models.

**Table S2**. Results of Linear Mixed-Effects Model predicting object dwell time without Text. Differences between groups were tested by including the relevant interaction terms. A beta estimate for a given interaction represents the estimated difference between children and adults. Main effects represent the average standardized estimates across both age groups Significant effects are reported in bold.

| **Fixed Effects** | **Description** | **B** | **SE** | ***t*** | ***P*** |
| --- | --- | --- | --- | --- | --- |
| **Intercept** | **Main effect** | **-0.20** | **0.05** | **-4.18** | **<0.001** |
| Age Group | Children-Adults | 0.04 | 0.05 | 0.68 | 0.49 |
| **Faces** | **Main effect** | **0.43** | **0.07** | **6.30** | **<0.001** |
| Age Group: Faces | Children-Adults | 0.07 | 0.06 | 1.21 | 0.23 |
| Touched | Main effect | 0.14 | 0.08 | 1.76 | 0.08 |
| **Age Group: Touched** | **Children-Adults** | **0.22** | **0.06** | **3.87** | **<0.001** |
| Hands | Main effect | -0.04 | 0.08 | -0.50 | 0.61 |
| **Age Group: Hands** | **Children-Adults** | **0.15** | **0.07** | **2.29** | **0.02** |
| Bodies | Main effect | -0.13 | 0.07 | -1.86 | 0.06 |
| **Age Group: Bodies** | **Children-Adults** | **0.07** | **0.06** | **1.33** | **0.18** |
| Eccentricity | Main effect | -0.05 | 0.03 | -1.77 | 0.08 |
| **Age Group: Eccentricity** | **Children-Adults** | **-0.10** | **0.02** | **-5.94** | **<0.001** |
| **Size** | **Main effect** | **0.25** | **0.02** | **10.56** | **<0.001** |
| Age Group: Size | Children-Adults | -0.03 | 0.02 | -1.80 | 0.07 |
| **GBVS** | **Main effect** | **0.08** | **0.03** | **3.05** | **0.002** |
| Age Group: GBVS | Children-Adults | 0.02 | 0.02 | 1.04 | 0.30 |

| **Random effects** |  |  |  |  | |  | |  | |  |
| --- | --- | --- | --- | --- | --- | --- | --- | --- | --- | --- |
| **Groups** | **Name** | ***SD*** | ***r*** |  | |  | |  | |  |
| Subjects | Intercept | 0.19 | Intercept | |  |  | |  | |  |
|  | Faces | 0.19 | -0.20 | Faces | | |  |  | |  |
|  | Touched | 0.13 | -0.32 | -0.37 | | Touched | | |  |  |
|  | Hands | 0.16 | -0.03 | -0.14 | | 0.90 | | Hands | |  |
|  | Bodies | 0.15 | -0.30 | 0.14 | | 0.77 | | 0.88 | | Bodies |
| Objects | Intercept | 0.42 | - |  | |  | |  | |  |
| Scenes | Intercept | 0.06 | - |  | |  | |  | |  |

**Table S3.** Results of binomial Generalized Linear Mixed-Effects Model predicting first fixations towards objects without text. Differences between groups were examined by including the relevant interaction terms. A beta estimate for a given interaction represents the estimated difference between children and adults. Main effects represent the average standardized estimates across both age groups Significant effects are reported in bold.

| **Fixed Effects** | **Description** | **B** | **SE** | **t** | **P** |
| --- | --- | --- | --- | --- | --- |
| **Intercept** | **Main effect** | **-4.83** | **0.21** | **-22.94** | **<0.001** |
| **Age Group** | **Children-Adults** | **-0.48** | **0.12** | **-4.09** | **<0.001** |
| **Faces** | **Main effect** | **2.27** | **0.26** | **8.73** | **<0.001** |
| Age Group:Faces | Children-Adults | -0.10 | 0.14 | -0.70 | 0.48 |
| Touched | Main effect | 0.53 | 0.33 | 1.62 | 0.11 |
| **Age Group:Touched** | **Children-Adults** | **0.98** | **0.21** | **4.79** | **<0.001** |
| Hands | Main effect | 0.49 | 0.32 | 1.53 | 0.12 |
| **Age Group:Hands** | **Children-Adults** | **0.54** | **0.20** | **2.75** | **0.01** |
| Bodies | Main effect | 0.07 | 0.27 | 0.25 | 0.80 |
| **Age Group:Bodies** | **Children-Adults** | **0.59** | **0.16** | **3.82** | **<0.001** |
| **Eccentricity** | **Main effect** | **-1.34** | **0.13** | **-10.49** | **<0.001** |
| **Age Group:Eccentricity** | **Children-Adults** | **-0.20** | **0.07** | **-3.10** | **0.002** |
| **Size** | **Main effect** | **1.78** | **0.13** | **13.40** | **<0.001** |
| Age Group:Size | Children-Adults | -0.09 | 0.07 | -1.39 | 0.16 |
| **GBVS** | **Main effect** | **0.78** | **0.13** | **5.99** | **<0.001** |
| Age Group:GBVS | Children-Adults | 0.05 | 0.06 | 0.86 | 0.39 |

| **Random effects** |  |  |  |  | |  | |  | |  |
| --- | --- | --- | --- | --- | --- | --- | --- | --- | --- | --- |
| **Groups** | **Name** | ***SD*** | ***r*** |  | |  | |  | |  |
| Subjects | Intercept | 0.04 | Intercept | |  |  | |  | |  |
|  | Faces | 0.29 | 0.88 | Faces | | |  |  | |  |
|  | Touched | 0.55 | -0.80 | -0.99 | | Touched | | |  |  |
|  | Hands | 0.41 | -0.99 | -0.82 | | 0.73 | | Hands | |  |
|  | Bodies | 0.35 | -0.98 | -0.77 | | 0.68 | | 1 | | Bodies |
| Objects | Intercept | 1.44 | - |  | |  | |  | |  |
| Scenes | Intercept | 0.67 | - |  | |  | |  | |  |

Figure S2. **Group differences in predicting object-directed fixations and dwell time without Text.** Bar plots depicting the beta estimates of a given object feature x Age interaction, which describe the difference in estimates between children and adults for a given object property. Panel a shows the interaction estimates for the LMM predicting object-directed dwell time and panel b depicts the binomial GLMM predicting first fixations towards objects after image onset across the included predictors Text, Faces, Touched, Hands and Bodies as well as Object-image centroid eccentricity (Ecc), (Object-) Size and graph-based visual salience (GBVS). Asterisks indicate the significance of a given group x predictor interaction. Results of model-based analyses that did not take into account the effect of text can be found in the supplementary materials. Error bars represent the 95% confidence intervals. *p < 0.05, **p < 0.01, ***p < 0.001.


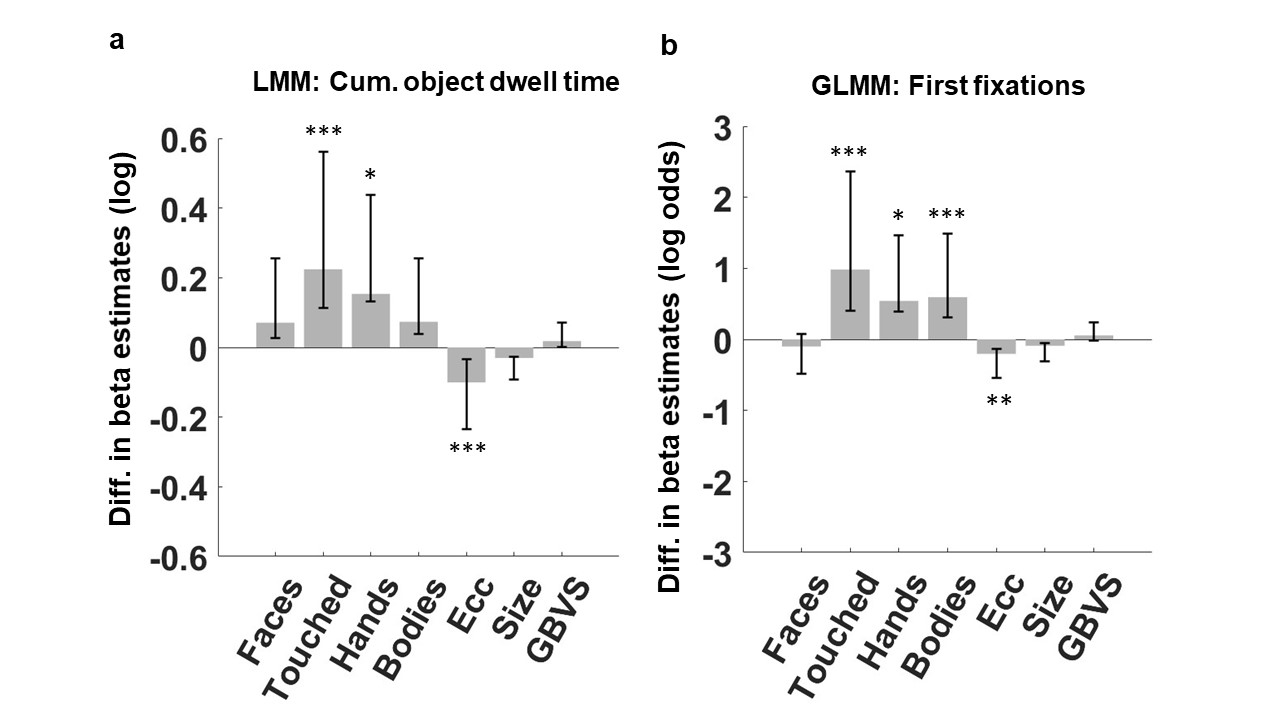


In the analysis predicting object-directed dwell time, significant interactions were found between *Age* and *Touched* and *Age* and *Hands*, indicating that when not controlled for *Text*, children devoted more dwell time towards *Touched* objects, *b* = 0.22, *t* = 3.87, *p* < 0.001 and *Hands*, *b* = 0.15, *t* = 2.29, *p* = 0.02, compared to adults. Furthermore, a significant interaction between *Age* and *Eccentricity* showed a stronger central bias for children than adults, *b* = -0.10, *t* = -5.94, *p* < 0.001. No significant differences in estimates were found for *Faces*, *b* = 0.07, *t* = 1.21, *p* = 0.23. Finally, we found no significant interactions between *Age* and object *Size* and *Age* and *GBVS*, indicating no significant differences between children and adults for the predictors *Size*, *b* = -0.03, *t* = 1.80, *p* = 0.07, and *GBVS*, *b* = 0.02, *t* = 1.04, p = 0.30.

When predicting whether an object was fixated immediately after image onset (first fixation), results showed significant interactions of *Touched*, *Hands*, and *Body* with *Age*. These interactions indicate that the probability of an object being fixated immediately after image onset was higher for *Touched*, *b* = 0.98, *t* = 4.79, *p* < 0.001, *Hands*, *b* = 0.54, *t* = 2.75, *p* = 0.01 and *Body*, *b* = 0.59, *t* = 3.82, *p* < 0.001, in children compared to adults. Further, a significant interaction between *Age* and *Eccentricity*, *b* = -0.20, *t* = -3.10, *p* = 0.002 indicated a stronger center bias for first fixations in children relative to adults. Finally, we found no significant interactions between *Age* and *Face*, *b* = -0.10, *t* = -0.70, *p* = 0.48, object Size, *b* = -0.09, *t* = 1.39, *p* = 0.16, and *GBVS*, *b* = 0.05, *t* = 0.86, *p* < 0.39.

These findings are in line with the stronger tendency to fixate *Touched* objects, *Hands* and *Bodies* observed for children (see simple inference statistics, Fig. 3 & 4, main text). At the same time, they suggest that these differences are mostly driven by the drastically reduced gaze bias towards *Text* in children. Non-significant interactions between *Age* and *Faces* when predicting object-directed dwell time and first fixations are not line with our earlier findings and hence might be explained by the additional control predictors included here, e.g. the stronger center bias in children. Also note, that the GLMM controlling for *Text* showed a stronger attraction of *Faces* for first fixations in adults, indicating that this effect is robust to low-level salience differences when controlling for the competing attraction to *Text* in adults.

**Group difference in gaze towards objects with implied motion**

Here we tested group differences in cumulative dwell time proportion towards objects with implied *Motion* using a permutation test. Fig S4 shows the distributions of cumulative dwell time proportion spent on objects with implied *Motion.*

Figure S3. **Group differences in gaze behaviour towards objects with implied *Motion***. Density plot showing the probability distributions of cumulative dwell time towards objects of the dimension *Motion* for children (red) and adults (blue). Data points depicted below indicate the individual dwell time proportion for *Motion*. Corresponding box plots above the data points show an overview of the summary statistics for each group and semantic category; the vertical line within a box represents the mean value. The left side of a box indicates the 25^th^ percentile and the right side the 75^th^ percentile. The whiskers represent the minimum and maximum values. **p* < 0.05, ***p* < 0.01, ****p* < 0.001 (Holm-Bonferroni corrected; see Methods)


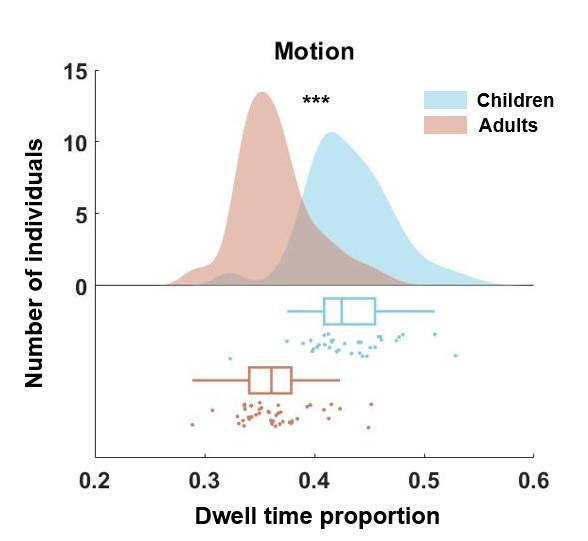


The findings showed that children devoted a significantly larger amount of their dwell time towards objects with implied *Motion* compared to adults, *t*obs = 8.01*, p* < 0.001. Note, for this analysis we calculated the dwell time proportion of fixations towards all objects, which are labelled *Motion*. As 92% of objects with implied *Motion* are also labelled *Hands*, *Bodies* or *Faces*, enhanced attentional biases towards objects of these dimensions in children may explain group differences shown for *Motion*.
